# Stage-specific cyanobacterial handoffs and heterotrophic subsidies underpin biocrust carbon accumulation

**DOI:** 10.1093/ismeco/ycag254

**Published:** 2026-09-04

**Authors:** Xian Zhang, Hua Li, Yingchun Han, Qi Li, Haijian Yang, Chunxiang Hu

**Affiliations:** CAS Key Laboratory of Algal Biology, Institute of Hydrobiology, Chinese Academy of Sciences, Wuhan 430072, China; College of Advanced Agricultural Sciences, University of the Chinese Academy of Sciences, Beijing 100049, China; CAS Key Laboratory of Algal Biology, Institute of Hydrobiology, Chinese Academy of Sciences, Wuhan 430072, China; CAS Key Laboratory of Algal Biology, Institute of Hydrobiology, Chinese Academy of Sciences, Wuhan 430072, China; Third Institute of Oceanography, Ministry of Natural Resources, Xiamen 361005, China; CAS Key Laboratory of Algal Biology, Institute of Hydrobiology, Chinese Academy of Sciences, Wuhan 430072, China; School of Life Sciences and Chemistry, Wuhan Donghu College, Wuhan 430212, China; CAS Key Laboratory of Algal Biology, Institute of Hydrobiology, Chinese Academy of Sciences, Wuhan 430072, China; CAS Key Laboratory of Algal Biology, Institute of Hydrobiology, Chinese Academy of Sciences, Wuhan 430072, China

**Keywords:** biological soil crusts, cyanobacterial inoculation, desert cyanobacteria, dryland restoration, life history strategies, microbial biomass carbon, metabolite exchange, syntrophy

## Abstract

Inoculating cyanobacteria is a widely adopted strategy to accelerate biocrust formation and restore dryland soil functions, yet the in situ mechanisms sustaining microbial biomass carbon (MBC) accumulation under seasonal stress remain poorly understood. Here, we combined a field inoculation experiment with multi-omic profiling (amplicon sequencing, metagenomics, metatranscriptomics, and metaproteomics) and genome-scale metabolic modeling to track the circannual development of induced cyanobacterial biocrusts in Hopq Desert, China. Although the inoculated *Konicacronema* strain (formerly *Microcoleus*) maintained significant taxonomic dominance, metatranscriptomic and metaproteomic data revealed a striking functional handoff. *Microcoleus*-like populations drove rapid initial biocrust formation via the oxidative pentose-phosphate pathway and enzymatic antioxidant systems, whereas *Oscillatoria* assumed transcriptional dominance during harsher seasonal transitions through enhanced light harvesting, cyclic electron transport, and non-enzymatic photoprotection. Genome-scale metabolic modeling predicted that dominant cyanobacteria acted primarily as net importers, receiving amino acids, vitamins, cell-wall precursors, and phosphate from heterotrophic partners, while supplying inorganic sulfur and iron–sulfur clusters. Co-occurrence networks and life-history analyses further revealed that MBC maintenance was associated with a shift from growth-oriented resource mobilization toward acquisition-oriented strategies mediated by ABC transporters. Structural equation modeling confirmed that solar radiation and soil salinity govern MBC indirectly by influencing cyanobacterial composition and the balance between phototrophic assimilation and resource-acquisition strategies. These findings demonstrate that biocrust carbon accumulation is sustained by temporal functional division among cyanobacterial guilds and metabolic subsidies from heterotrophic partners, rather than by the persistent activity of a single pioneer strain, and provide a trait-based roadmap for designing resilient, multifunctional synthetic inocula for dryland restoration.

## Graphical abstract

**Graphical abstract:**
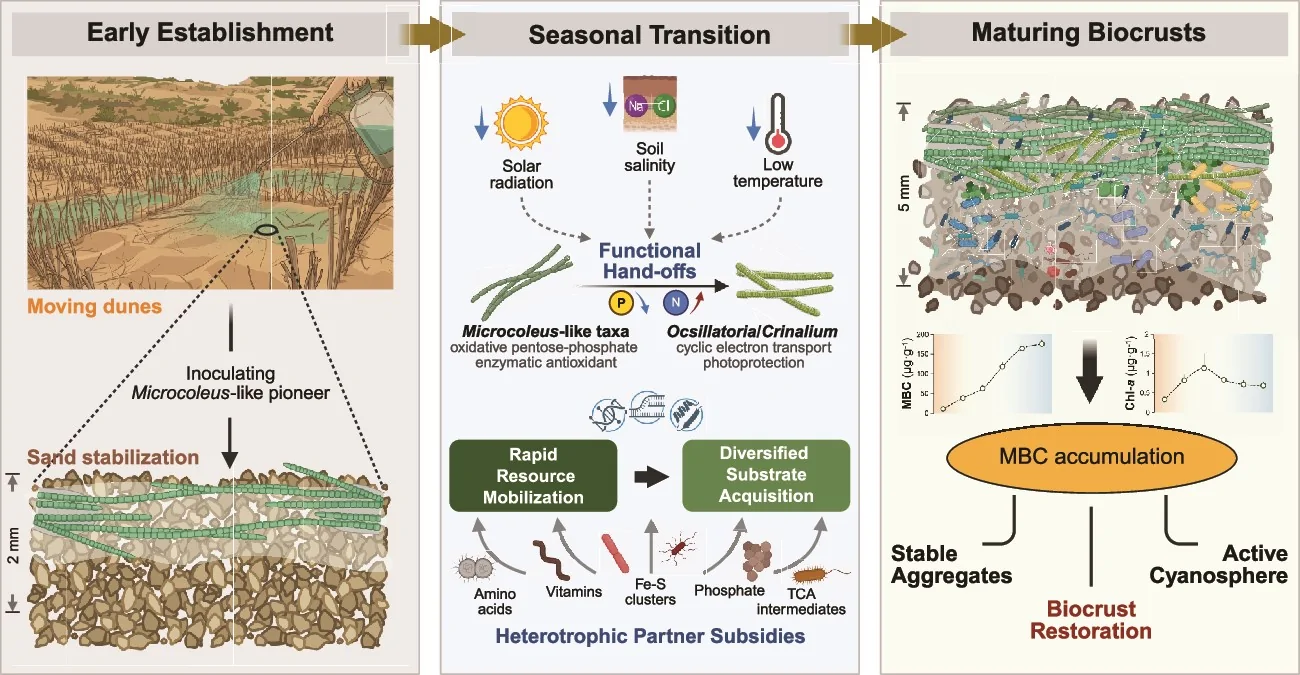

## Introduction

Drylands, which cover 46% of the global terrestrial surface, face intensifying threats from erosion and degradation in an era of escalating climate change, yet over 30% of their exposed soils are naturally armored by biological soil crusts (i.e*.* biocrusts) [1, 2]. These delicate micro-ecosystems originate from cyanobacterial colonization, which stabilizes sand surfaces and facilitates the establishment of other cryptogams and soil microfauna, ultimately forming cohesive upper layers that mitigate wind and water loss [3, 4]. By enhancing water retention, improving soil fertility, and fostering belowground biotic activity [4, 5], biocrusts provide indispensable ecosystem functions in arid regions [6, 7]. However, their slow natural development has prompted the exploration of cyanobacterial inoculation as a novel strategy to accelerate biocrust formation, offering a promising and sustainable path for land restoration in dryland ecosystems [8, 9].

Among pioneer cyanobacteria, filamentous bundle-forming strains (historically identified as *Microcoleus*, particularly *M. vaginatus*) are keystone species in biocrusts because of their global ubiquity and proven efficacy in dryland restoration [10–12]. These photoautotrophs rapidly stabilize shifting sands and ameliorate soil structure, promoting vascular plant establishment [13]. Importantly, they secrete extracellular polymeric substances (EPS) that sustain a specialized heterotrophic cyanosphere, orchestrating syntrophic metabolic networks essential for biocrust maturation and resilience [14]. Despite evidence of their long-term dominance in inoculated biocrusts, extending up to 15 years post-introduction [13], advances in polyphasic taxonomy have reclassified many former *Microcoleus* taxa into distinct genera such as *Coleofasciculus*, *Funiculus*, *Konicacronema*, and *Symplocastrum* [15, 16]. This taxonomic flux highlights critical unresolved questions about competitive dynamics between introduced strains and indigenous relatives, as well as the metabolic adaptations and resource-sharing strategies that enable their success in extreme arid habitats [17, 18].

While maximizing biomass stock is the fundamental aim of biocrust engineering [19], its performance often depends on microhabitat conditions and is governed by the intrinsic trade-off among microbial growth, resource acquisition, and stress tolerance [20, 21], conceptualized through the life-history strategy [22]. In the **Y**ield-**A**cquisition-**S**tress (Y-A-S) framework, a predominance of Y- or A-strategies typically favors biomass accumulation, whereas shifts toward the S-strategy under harsh conditions result in lower net yields [21, 22]. These strategy shifts serve as sensitive indicators of community-level responses to environmental pressures, such as nutrient stresses or fluctuating carbon cycling rates [23, 24]. In dryland biocrusts, where carbon fixation is rigorously modulated by salinity and aridity gradients [25], “*Microcoleus*” navigates resource scarcity via an integrated interactome, involving direct nutrient uptake alongside carbon-nitrogen trades and phosphorus provisioning from sheath-associated heterotrophic symbionts [26, 27]. Although recent multi-omics and metabolic modeling have begun to reveal intricate interdependencies, including the reciprocal exchange of vitamins and amino acids under abiotic stress [17, 28], critical knowledge gaps remain concerning the temporal dynamics of cyanobacterial consortia and causal linkages between specific metabolic interactions and community-level MBC in natural settings.

**Figure 1:**
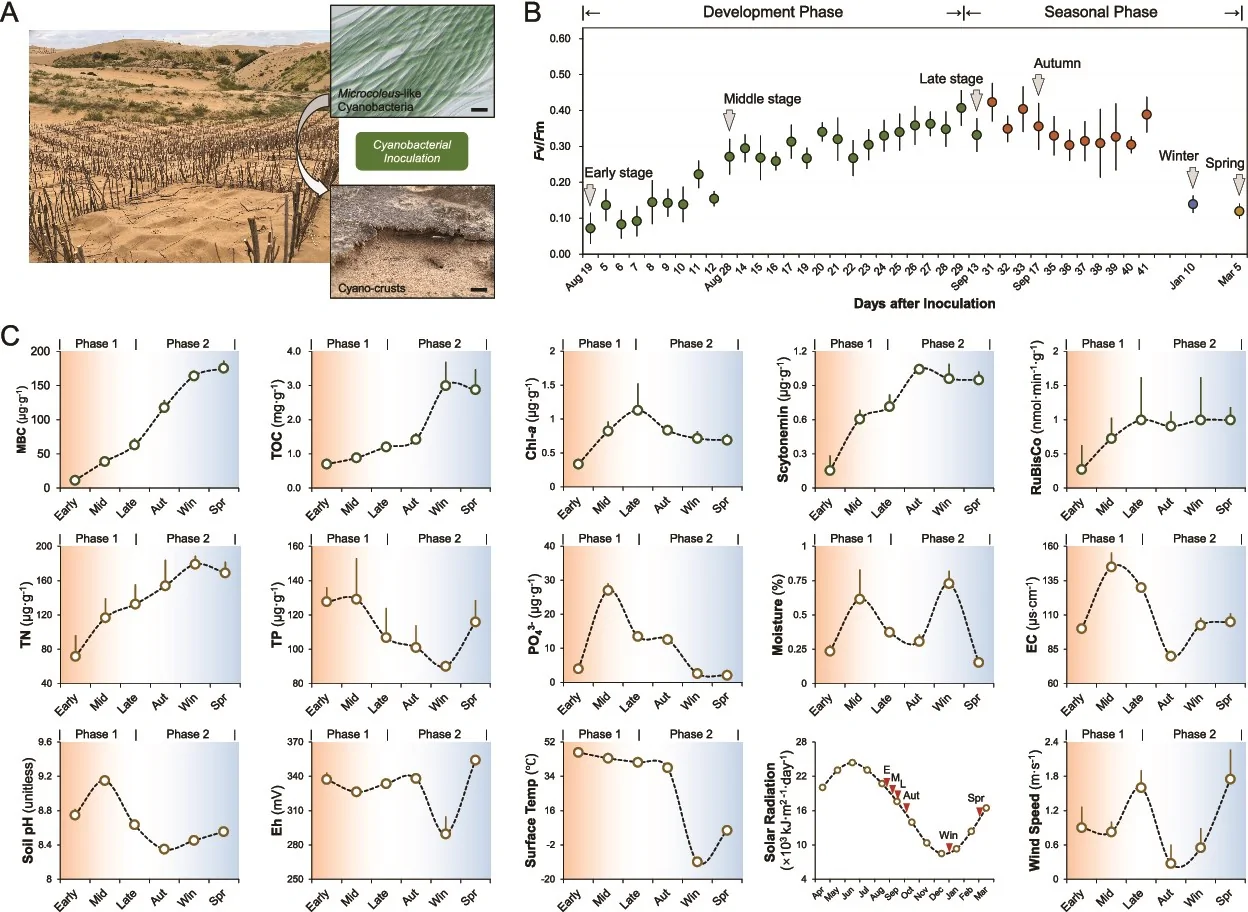
Environmental context and edaphic trajectories during the succession of inoculated cyanobacterial biocrusts. (A) Site overview, microscopic morphology of the inoculated *Konicacronema* sp. APE-1 strain, and macroscopic progression of the induced biocrusts from moving sand to a cohesive surface crust; (B) temporal dynamics of the maximum quantum yield of PSII (*F*v/*F*m) across the summer establishment and subsequent seasonal stages (mean ± standard error); (C) Circannual trajectories of carbon and nutrient pools, edaphic properties, and meteorological drivers. MBC, microbial biomass carbon; TOC, total organic carbon; TN, total nitrogen; TP, total phosphorus; PO_4_^3−^, phosphate; EC, salinity; Eh, redox potential; Chl-*a*, chlorophyll *a*; Scy, scytonemin; RuBisCO, ribulose-1,5-bisphosphate carboxylase/oxygenase; Temp, surface temperature. Sampling stages are denoted as Early, Middle, and Late for the summer development phase, and Aut (Autumn), Win (Winter), and Spr (Spring) for the seasonal phase.

To address these concerns, we conducted an *in-situ* experiment in the Hopq Desert, inoculating native moving dunes with a high-exopolysaccharide-producing cyanobacterial strain, *Konicacronema* sp. (Gomontiellales, a member of the *Microcoleus*-like complex) [29, 30] (Fig. 1A). By integrating high-throughput amplicon sequencing with multi-omic approaches (i.e. metagenomics, metatranscriptomics, and metaproteomics), edaphic physicochemical profiling, and meteorological trace, we tracked biocrust development dynamics across seasonal shifts over an approximate annual cycle. We sought to (i) provide an in-depth assessment of the temporal trajectory of inoculated cyanobacterial abundance, metabolic activity, and life-history strategies, with a particular focus on identifying the active fraction of the cyanosphere during metabolic state transitions [31, 32]; (ii) employ co-occurrence networks, metagenome-assembled genomes (MAGs), and *in-silico* metabolic modeling to map metabolite exchanges between the cyanobacterial edificator and its associated microbiota [33, 34], thereby linking “*Microcoleus*”-driven proliferation to metabolic complementarity and MBC dynamics; and (iii) unravel the multi-scale regulatory mechanisms of carbon sequestration in biocrusts, through the cascading effects of environmental predictors, cyanobacterial inoculum, and microbial life-history strategies. To our knowledge, this study represents the first circannual multi-omic scrutiny of biocrust-forming processes in the real world. Our findings offer novel insights into the survival strategies of dryland extremophile consortia and provide a theoretical foundation for optimizing microbial inoculation techniques in global land restoration practices.

## Materials and methods

### Cyanobacterial inoculation and biocrust sampling

We isolated and purified the pioneering cyanobacterium *Konicacronema* sp. (formerly classified as *Microcoleus* sp.) from indigenous biocrusts [30]. A high-performing strain, selected for rapid biomass accumulation and prolific EPS secretion, was mass-cultivated and inoculated uniformly onto shifting sand surfaces at the experimental site in Inner Mongolia, China. The initial inoculum density was standardized to ~5 μg Chl *a*·cm^−2^. To mitigate wind erosion and sand burial, we established a straw-checkerboard barrier before inoculation. For the first month after inoculation, biocrusts were mist-sprayed twice daily (morning and evening) to promote initial colonization. Samples were collected on Days 4, 13, and 29 post-inoculation to capture early development dynamics, and in September 2018 (Autumn), January 2019 (Winter), and March 2019 (Spring) to encompass seasonal transitions. Subsamples for physicochemical analyses were air-dried in desiccators, whereas those for multi-omics were flash-frozen and stored at −80°C.

### Determination of edaphic physicochemical properties

Soil water content was determined gravimetrically immediately upon arrival in the laboratory. Biocrust samples were then ground and passed through a 180-μm sieve. Chlorophyll *a* (Chl *a*) and phycoerythrin were extracted in 100% acetone and quantified spectrophotometrically. Soil pH, redox potential (Eh), and electrical conductivity (salinity proxy) were measured in 1:5 (w/v) soil-to-deionized water suspensions using calibrated electrodes [35]. Total organic carbon (TOC) was analysed with an elemental analyzer (Vario TOC/TN_b_ Select analyzer, Elementar, Germany) after acid removal of carbonates [6]. MBC was determined by the chloroform fumigation-extraction method [36]. Total nitrogen (TN) and total phosphorus (TP) were quantified by alkaline and potassium persulfate digestion, respectively, followed by colorimetric analysis. Soluble phosphate (PO_4_^3−^) was measured by ion chromatography (Dionex ICS-5000+, Thermo Fisher Scientific, USA). Ribulose-1,5-bisphosphate carboxylase/oxygenase (RuBisCO) activity was assayed with a commercial spectrophotometric kit (Bioss, China). Chlorophyll fluorescence parameters were recorded with a Handy PEA fluorometer (Hansatech, UK) after 10 min of dark adaptation using a saturating pulse of 1500 μmol photons m^−2^·s^−1^.

### Nucleic acid extraction and amplicon sequencing

Total RNA was extracted with an RNeasy PowerSoil Total RNA Kit (Qiagen, USA). Residual DNA was further removed from the isolated RNA with the DNase Max Kit. After RNA elution, DNA was recovered from the same column using the RNeasy PowerSoil DNA Elution Kit. We assessed RNA concentration and purity using a NanoDrop 2000c spectrophotometer (Thermo Fisher Scientific, USA), and evaluated its integrity using an Agilent 2100 Bioanalyzer (Agilent Technologies, USA). The quality of DNA was evaluated by agarose gel electrophoresis, and its concentration was quantified using the PicoGreen assay. The V3-V4 region of the bacterial 16S rRNA gene was amplified with the primers 338F (5’-ACT CCT ACG GGA GGC AGC A-3′) and 806R (5′-GGA CTA CHV GGG TWT CTA AT-3′) [37]. We purified, quantified, pooled equimolarly, and sequenced the polymerase chain reaction (PCR) products on the Illumina MiSeq PE300 platform. Raw reads were quality-filtered with *fastp* v0.14.1, merged with *FLASH* v1.2.7 [38, 39], and then processed through the *DADA2* pipeline for denoising, dereplication, and chimera removal [40]. Taxonomic assignment was performed against the *SILVA* v138 database using the *RDP classifier* v2.14. The amplicon sequence variant (ASV) table was rarefied to a minimum of 14 000 sequences per sample to normalize sequencing depth.

### Metagenomic and metatranscriptomic sequencing and assembly

Metagenomic libraries were prepared from fragmented genomic DNA (~ 300 bp) using the TruSeq™ DNA Sample Prep Kit and sequenced on the Illumina NovaSeq 6000 platform. Reads were quality-filtered and trimmed using *SeqPrep* and *Sickle*. For metatranscriptomics, rRNA was depleted using the Ribo-Zero rRNA Removal Kit, and the remaining mRNA was reverse-transcribed into cDNA, which was then used to construct a library and sequenced on the Illumina HiSeq X Ten platform. Residual rRNA reads were further removed with *SortMeRNA* [41]. We used a hybrid assembly strategy to maximize coverage and accuracy. Metagenomic reads were first assembled with *MEGAHIT* and refined with *Newbler* for secondary assembly [42]. Metatranscriptomic reads were assembled with *Trinity* v2.15.1 [43], retaining transcripts ≥300 bp. Open reading frames were predicted using *MetaGene* and *TransGeneScan* v1.3.0 [44, 45]. Non-redundant gene catalogs were generated separately for the metagenomic and metatranscriptomic datasets by clustering at 95% identity and 90% coverage with *CD-HIT* v4.8.1 [46]. Thus, the two catalogs were processed independently and not merged. Metagenomic gene abundance and metatranscriptomic expression levels were quantified by mapping reads back to their respective non-redundant catalogs with *SOAPaligner* [47] and normalized to transcripts per million (TPM) by correcting for gene length and total mapped reads per sample. We annotated taxonomy by aligning sequences against the NCBI non-redundant protein database using *BLASTP* v2.3.0 (e-value ≤1e-5). Functional annotation was performed independently for each catalog using *BLASTP* (e-value ≤1e-5) against the Kyoto Encyclopedia of Genes and Genomes (KEGG) database. KEGG Orthology (KO) identifiers and pathway assignments were retained from the best-hit annotations.

### Metagenome-assembled genome recovery and quality filtering

Metagenomic reads were binned, refined, and reassembled using the *metaWRAP* v1.3.2 pipeline [48]. The MAG quality was assessed with *CheckM* v1.0.18 [49]. In total, 533 MAGs were recovered using thresholds of >50% completeness and < 10% contamination. Of these, 75 high-quality MAGs (>80% completeness, <5% contamination) were retained for genome-scale metabolic modeling and downstream genome-resolved analyses. Taxonomic classification was performed with *GTDB-Tk* v1.5.1 against the Genome Taxonomy Database release R202 [50]. We further placed quality-filtered cyanobacterial MAGs in context with reference genomes of bundle-forming *Microcoleus* spp. sourced from the NCBI database and our laboratory collection [51]. Relative abundance and transcriptional activity of each MAG were quantified using *CoverM* v0.7.0 and expressed as TPM [52]. We calculated average nucleotide identity (ANI) to reference genomes using *fastANI* v1.34 at a 95% threshold [53]. We profiled functional genes against the KEGG database and classified the metabolic potential of key MAGs using the **Y**ield-**A**cquisition-**S**tress-**P**ersistence (Y-A-S-P) framework to evaluate and compare the life-history strategies governing community assembly and biomass maintenance [22].

### Metaproteomic analysis and proteogenomic integration

We extracted proteins from biocrust samples, and those that met stringent quality criteria were reduced and alkylated. Standardized protein concentrations were then digested with trypsin, and the resulting peptides were quantified with a Fluorometric Peptide Quantification Kit (Thermo-Fisher Scientific, USA). For mass spectrometry, equivalent peptide loads were reconstituted in loading buffer (0.5 μg·μl^−1^) and analysed on a Q-Exactive Plus Orbitrap Mass Spectrometer (Thermo Fisher Scientific, USA) coupled with an ultra-performance liquid chromatography system. Chromatographic separation was performed over a 120-min gradient to ensure comprehensive peptide detection. To enable accurate protein identification from complex environmental samples, we constructed a customized reference protein database by merging non-redundant gene sequences from the metagenomic and metatranscriptomic datasets, clustering at 98% identity and 100% coverage with *CD-HIT* v4.8.1 [46], and selecting the longest representative sequence per cluster. We searched spectra against this proteogenomic database using *Proteome Discoverer* v2.4 (Thermo-Fisher Scientific, USA). We then transferred taxonomic and functional annotations of the identified proteins from the corresponding pre-annotated entries in the customized proteogenomic database. For taxonomic annotation, protein sequences were searched against the NCBI non-redundant protein database using *BLASTP* v2.3.0 with an e-value threshold of ≤1e-5, and the annotation corresponding to the best significant hit was retained. Functional annotation was performed against the KEGG database using *BLASTP* with the same e-value threshold, and the KEGG Orthology identifier and pathway assignment corresponding to the best significant hit were retained. Given the limited taxonomic resolution of highly conserved proteins, we interpreted protein-based taxonomic assignments, particularly at the genus and species levels, cautiously and used them primarily as supporting evidence alongside the corresponding metagenomic and metatranscriptomic results.

### Metabolic complementarity and cross-feeding predictions

To evaluate potential metabolic dependencies within the biocrust community, we reconstructed genome-scale metabolic models using *CarveMe* v1.4.1 [54] for the 75 high-quality MAGs (>80% completeness, <5% contamination). Metabolic complementarity between MAG pairs was assessed using *PhyloMint* [33], which quantifies the extent to which the capacity of one genome compensates for biosynthetic gaps in another. Because microbial interactions are direction-dependent, we calculated asymmetric complementarity scores to distinguish potential metabolite donors from recipients. We identified high-complementarity interactions using a random-matrix-theory threshold [55]. Stage-specific principal cyanobacteria-heterotroph associations were selected based on co-occurrence network modules identified in the metatranscriptomic networks (see below). *SMETANA* v1.2.0 [56] was then applied to these selected pairs to predict putative exchanged metabolites and the directionality of metabolic dependencies.

### Statistical analyses

All statistical analyses and visualizations were performed in *R* v4.2.0. Temporal trends in edaphic properties and community metrics were visualized with the *tidyverse*, *plotrix*, and *ggh4x* packages. We evaluated relationships among community structure, functional traits, and environmental variables using Mantel tests and Kendall rank correlations. To identify the most influential predictors of MBC, we fit random forest models using the *randomForest* package. Piecewise structural equation modeling (pSEM) was used to trace causal pathways from environmental drivers through cyanobacterial activity and life-history strategies to MBC using the *piecewiseSEM* package. To account for temporal dynamics, we incorporated differences between adjacent sampling points into the matrices to capture rates of change in pSEM. Differential transcriptional activity across the six sampling stages was assessed using *ZicoSeq* (default parameters) on the genus-level metatranscriptomic abundance matrix. Low-abundance genera were not removed before genus-level analysis. The genera showing significant shifts were determined at a false discovery rate-adjusted *P*-value <0.05. For phylogenetic reconstruction, we inferred maximum-likelihood trees from concatenated single-copy protein sequences using IQ-TREE and refined them in *iTOL*. Co-occurrence networks were constructed separately for the development (Early–Middle–Late) and seasonal (Autumn–Winter–Spring) periods. Input matrices comprised genus-level metatranscriptomic abundances (genera >0.01% retained), metabolic pathway expression profiles, and measured physicochemical variables. Pairwise Spearman correlations were calculated, and edges were retained at |*ρ*| >0.6 and adjusted *P* < 0.05. We visualized the networks in *Gephi*, and the modularity analysis function identified topological modules. Dominant cyanobacteria-heterotroph associations within each module were used to guide the selection of pairs for *SMETANA* analysis.

## Results

### Dynamics of physicochemical properties and meteorological context

Successful cyanobacterial colonization and subsequent maturation of the induced biocrust were characterized by distinct shifts in photochemical efficiency and nutrient stoichiometry (Fig. 1). Photosynthetic performance indicators, specifically the maximum quantum yield of PSII (*F*v/*F*m) and Chl *a* content, showed a robust upward trajectory during the initial development phase (Early to Late stages), followed by a significant decline during the seasonal transitions, with the lowest values recorded in the Winter and Spring stages (Fig. 1B and C). In contrast, markers of UV protection (scytonemin), carbon sequestration (TOC and MBC), and nitrogen accumulation (TN) increased steadily throughout the circannual cycle. Soluble phosphate (PO_4_^3−^) concentrations were progressively depleted, reaching their lowest levels during the colder months. These edaphic trends closely tracked seasonal meteorological fluctuations (Fig. 1C). The Winter stage had minimal solar radiation and surface temperatures, whereas the Spring stage had the highest wind speeds and thus represented the most severe environmental stress for the biocrust community.

### Successional dynamics of the biocrust community

**Figure 2:**
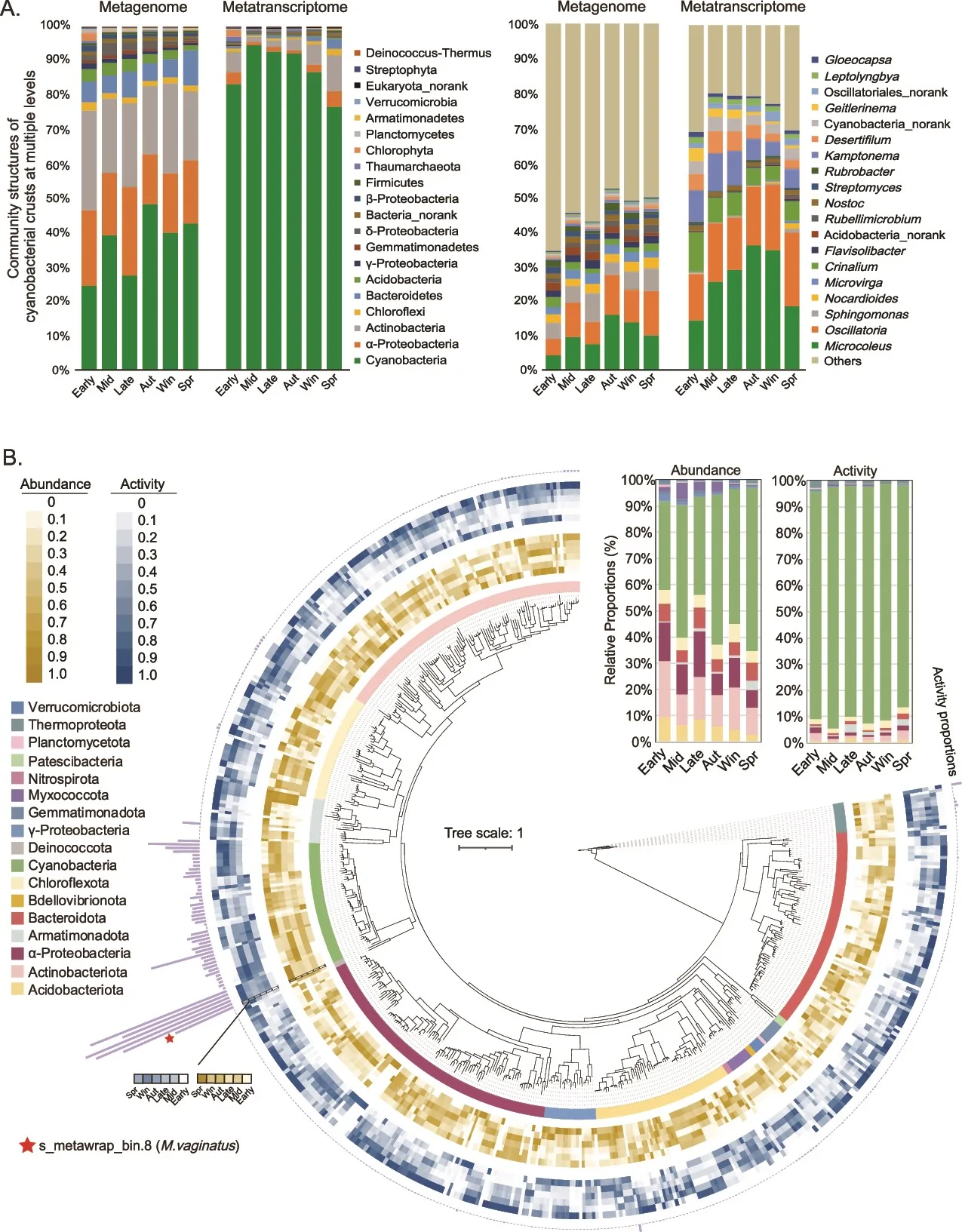
Decoupling of genomic potential and transcriptional activity during cyanobacterial biocrust succession. (A) Phylum- and genus-level community composition derived from metagenomic (DNA-based genomic potential, left) and metatranscriptomic (RNA-based transcriptional activity, right) datasets. The marked transition from *microcoleus*-like transcriptional dominance during early establishment to *Oscillatoria*-associated activity during the seasonal phase illustrates the functional handoff among cyanobacterial guilds. (B) Taxonomic classification and relative abundance of the 533 metagenome-assembled genomes (MAGs). Inner rings denote taxonomic hierarchy, and outer heatmaps display normalized abundance (metagenomic) and transcriptional activity (metatranscriptomic) profiles for each MAG. The contrast between the two outer rings highlights inconsistencies in the soil community across phases of primary succession.

**Figure 3:**
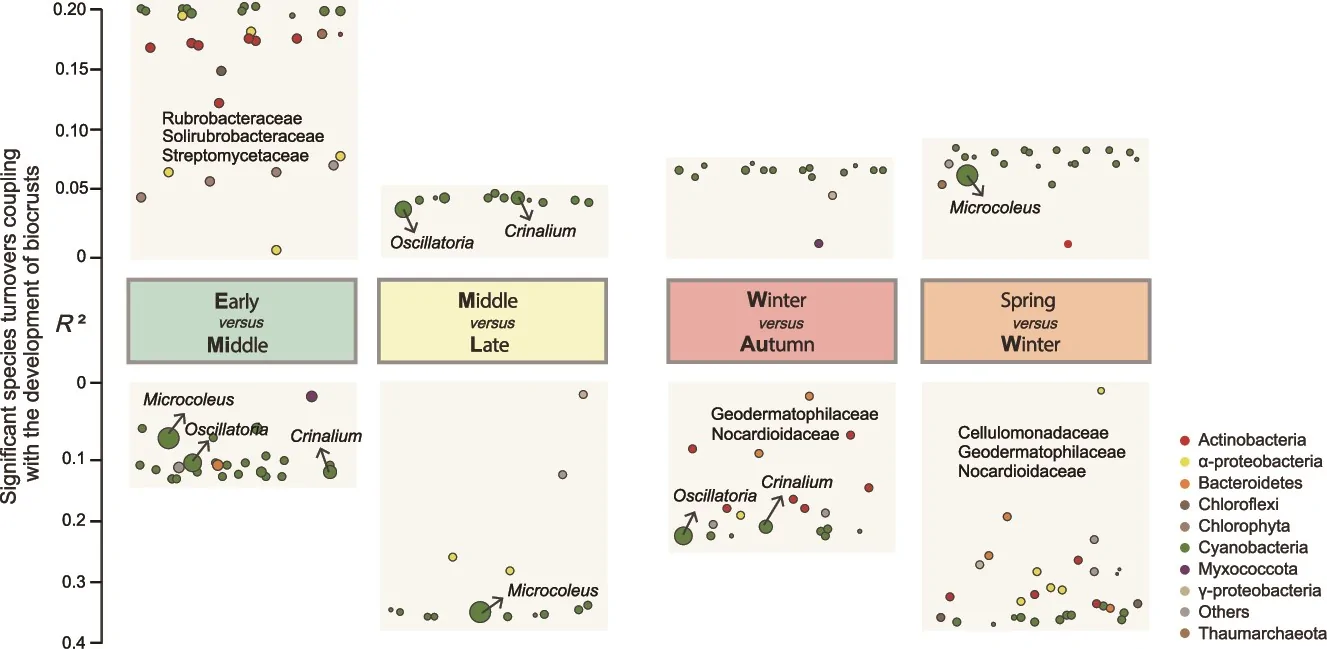
Stage-specific shifts in transcriptional activity among dominant microbial genera. *ZicoSeq* analysis of metatranscriptomic data highlighted genera with significant differential transcriptional activity across the six sampling stages (Early, Middle, and Late for the development phase, and Autumn, Winter, and Spring for the seasonal phase). Key cyanobacterial contributors (*Microcoleus*-like taxa, *Oscillatoria*, and *Crinalium*) and major heterotrophic Actinobacterial families are explicitly annotated. Node colors correspond to distinct taxonomic lineages.

High-resolution amplicon sequencing identified ASV10, corresponding to the inoculated cyanobacterium *Konicacronema* sp. APE-1 (*Microcoleus*-like clade), as the primary pioneer taxon. Its relative abundance increased steadily during the initial development phase, reached approximately 34% in the Spring stage, and exhibited characteristic fluctuations during the intervening Autumn and Winter stages (Fig. S1). Integrated multi-omic profiling confirmed that Cyanobacteria remained the most abundant and transcriptionally active phylum throughout the study period. Notably, although metagenomic data indicated a continuous increase in cyanobacterial genomic potential, metatranscriptomic analysis revealed a distinct decline in overall transcriptional activity during spring. At the genus level, metatranscriptomic profiles showed that *Microcoleus*-like taxa and *Oscillatoria* dominated the early development stages, whereas *Oscillatoria* sustained an increasing activity trend across subsequent seasonal transitions (Fig. 2A). Metaproteomic profiles were broadly consistent with early-stage cyanobacterial dominance (Fig. S2), but the seasonal increase in *Oscillatoria* activity was resolved primarily by the metatranscriptomic data. To mitigate potential biases arising from data compositionality, we employed *ZicoSeq* to identify genera with significant differential transcriptional activity across the six sampling stages (Fig. 3). During the initial development phase, *Microcoleus*-like activity was significantly up-regulated, while *Oscillatoria*, *Crinalium*, and specific Actinobacterial families (Rubrobacteraceae, Soilrubrobacteriaceae, and Streptomycetaceae) were significantly down-regulated. This pattern reversed during the seasonal phase, when *Oscillatoria* (~20% relative abundance), *Crinalium* (~7%), and a distinct suite of Actinobacteria (Geodermatophilaceae, Nocardioidaceae, and Cellulomonadaceae) showed marked transcriptional dominance, while *Microcoleus*-like activity declined. Because metatranscriptomics more directly reflects physiological states than metagenomic potential or metaproteomic abundance, we used the metatranscriptomic dataset as the primary input for subsequent functional and metabolic analyses.

### Metabolic shifts in cyanobacteria-dominated communities

**Figure 4:**
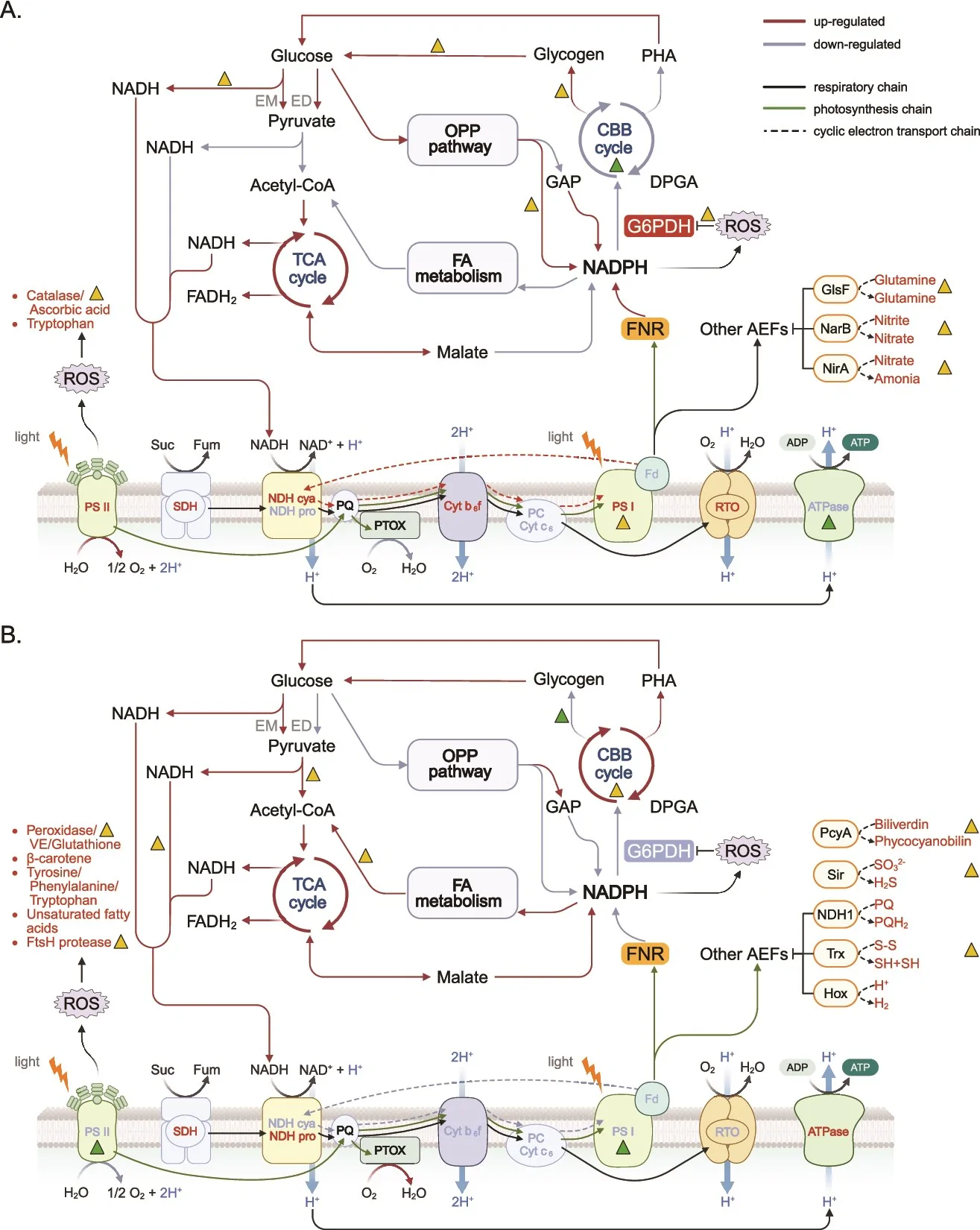
Functional transition from growth-oriented to stress-resilient metabolism across biocrust succession. Metabolic pathway maps illustrating significantly up-regulated (red arrows) and down-regulated (blue arrows) processes or genes during the (A) development phase and (B) seasonal transition phase, based on metatranscriptomic profiling. Metaproteomic validation is indicated by triangles, where yellow denotes up-regulation and green denotes down-regulation. Black lines represent the respiratory chain, green lines the linear photosynthetic electron transport chain, and black dashed lines the cyclic electron transport chain. CBB, Calvin-Benson-Bassham cycle; DPGA, 1,3-diphosphoglycerate; ED, Entner-Doudoroff pathway; EM, Embden-Meyerhof-Parnas pathway; FNR, ferredoxin-NADP+ oxidoreductase; Fum, fumarate; G6PDH, glucose-6-phosphate dehydrogenase; GAP, glyceraldehyde-3-phosphate; OPP, oxidative pentose phosphate pathway; PHA, polyhydroxyalkanoates; Suc, succinate; TCA, tricarboxylic acid cycle.

Metatranscriptomic profiling revealed a clear functional transition as the biocrusts shifted from initial formation to seasonal maturation (Fig. 4). During the developmental phase, cyanobacterial expansion was accompanied by significant up-regulation of pathways supporting primary production and rapid growth, including glycogen synthesis, the oxidative pentose-phosphate and Entner-Doudoroff (ED) pathways, assimilatory nitrogen and sulfur reduction, enzymatic antioxidant systems, and ascorbate (vitamin C) biosynthesis (Fig. 4A). In contrast, the seasonal phase was characterized by broad down-regulation of these biosynthetic processes, with the notable exceptions of the Calvin–Benson–Bassham (CBB) cycle, light-harvesting components (eg, phycocyanobilin:ferredoxin oxidoreductase, PcyA), and cyclic electron transport, which remained active (Fig. 4B). By the Spring stage, the community exhibited signs of metabolic quiescence, reflected in a significant decrease in glucose-6-phosphate dehydrogenase activity, a proxy for reduced metabolic flux. Metaproteomic data (yellow triangles in Fig. 4) corroborate these transcriptional patterns at the functional protein level.

At the genus level, the observed metabolic dynamics were associated with clear successional turnover in taxonomic dominance (Fig. S3). *Microcoleus*-like populations primarily drove the rapid growth and enzymatic antioxidant signatures characteristic of the development phase. In contrast, the functional profile of the seasonal phase was dominated by *Oscillatoria*, which sustained metabolic activity through non-enzymatic ROS scavenging (β-carotene, vitamins C and E), assimilatory sulfate reduction, and enhanced light harvesting. Notably, the capacity of *Oscillatoria* to maintain cyclic electron transport and light-harvesting function during the harsh Spring stage enabled it to outcompete *Microcoleus*-like taxa in terms of transcriptional activity. These contrasting functional profiles indicate that *Microcoleus*-like populations prioritized rapid biomass accumulation via oxygenic photosynthesis and efficient carbon storage during early development, whereas *Oscillatoria*-dominated seasonal stages relied on specialized stress-tolerance mechanisms and flexible energy acquisition to maintain activity under seasonal stress.

### Metabolic modular architecture and life-history strategies

**Figure 5:**
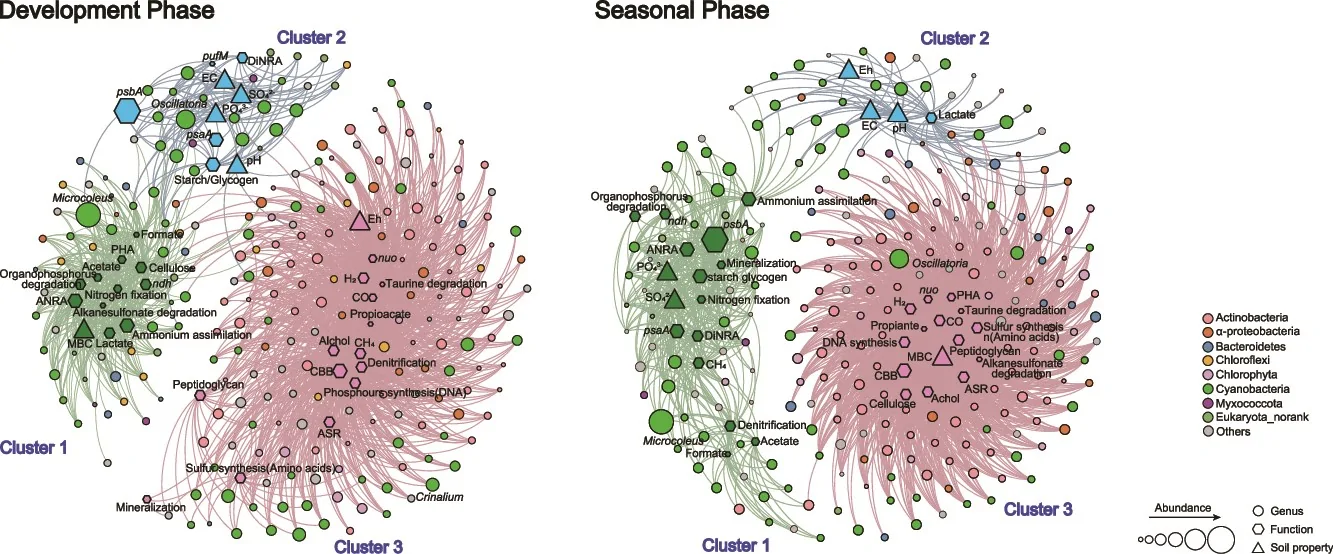
Co-occurrence networks linking microbial taxa, metabolic functions, and edaphic properties. Networks are constructed from metatranscriptomic data for the development (left) and seasonal (right) phases. Nodes represent bacterial genera (circles, >0.01% relative abundance), metabolic functions (hexagons), and physicochemical parameters (triangles). Node sizes are proportional to relative abundance, functional expression level, or parameter magnitude. Edges represent significant spearman correlations (*ρ* > 0.6, adjusted *P* < .05). Circle colors denote phylum-level taxonomy (class-level for Proteobacteria).

Co-occurrence networks were constructed to delineate interactions among transcriptionally active taxa, metabolic pathways, and edaphic properties during the development and seasonal phases (Fig. 5). In both phases, the networks resolved into three distinct topological modules. During the initial development phase, the module strongly associated with MBC (Cluster 1) linked *Microcoleus*-like taxa with Bacteroidetes and was enriched in pathways for assimilatory nitrite reduction and the mobilization of organic carbon, phosphorus, and sulfur. In contrast, Cluster 3 was dominated by *Crinalium* and diverse Actinobacteria and primarily harbored the CBB cycle, assimilatory sulfate reduction, and various chemolithotrophic functions.

During the seasonal phase, the network topology shifted markedly. The *Microcoleus*-associated module (Cluster 1) co-clustered with Myxococcota and was enriched in phototrophic energy acquisition and nitrate reduction pathways. Notably, the primary MBC-associated hub moved to Cluster 3, which was dominated by *Oscillatoria* and Actinobacteria and encompassed the CBB cycle and assimilatory sulfate reduction. When we examined these topological patterns through the Y-A-S-P life-history strategy framework, MBC-associated modules in both phases were consistently enriched in acquisition-oriented (A-strategy) pathways, particularly ABC transporter systems (Figs. S4, S5). Although baseline transport capacities for ferric iron, phosphorus, and glutamate remained relatively stable, stage-specific specializations were observed in which *Microcoleus*-associated modules prioritized fructose and sulfur transport during development, whereas *Oscillatoria*-associated modules favored neutral amino acid transport during the seasonal transition (Fig. S6). In contrast, non-MBC-associated modules (*Crinalium*-Actinobacteria and *Microcoleus*-Myxococcota) exhibited a phototrophy-oriented (P-strategy) profile. Across the entire dataset, community-level MBC was positively correlated with the P/A-strategy ratio (Fig. S7). These patterns indicate that the observed shift in MBC-associated modules from *Microcoleus*- to *Oscillatoria*-dominated configurations coincided with a strategic transition from rapid resource mobilization toward diversified substrate acquisition mediated by specialized ABC transporters.

### Syntrophic partnerships and metabolic exchange

To examine potential metabolic interdependencies among co-occurring and co-active taxa in the biocrust community, we integrated MAG-resolved metabolic modeling with the stage-specific partnerships identified by network analysis and *ZicoSeq* (Fig. 3). Among the recovered MAGs, *s_matewrap_bin.8* was identified as a key edificator population based on high phylogenetic similarity to *Microcoleus vaginatus* (Fig. 2B, Fig. S8) and elevated abundance and transcriptional activity across the metagenomic and metatranscriptomic datasets. Genome-scale metabolic modeling with *SMETANA* confirmed that the most robust complementary interactions occurred between particular MAG pairs of cyanobacteria and heterotrophic bacteria (Fig. S9). These predicted interactions were in concert with the successional partnerships resolved by *ZicoSeq*, namely *Microcoleus*-Bacteroidetes and *Crinalium*-Actinobacteria associations during the development phase, and *Microcoleus*-Myxococcota and *Oscillatoria*-Actinobacteria associations during the seasonal phase (Fig. 3).

**Figure 6:**
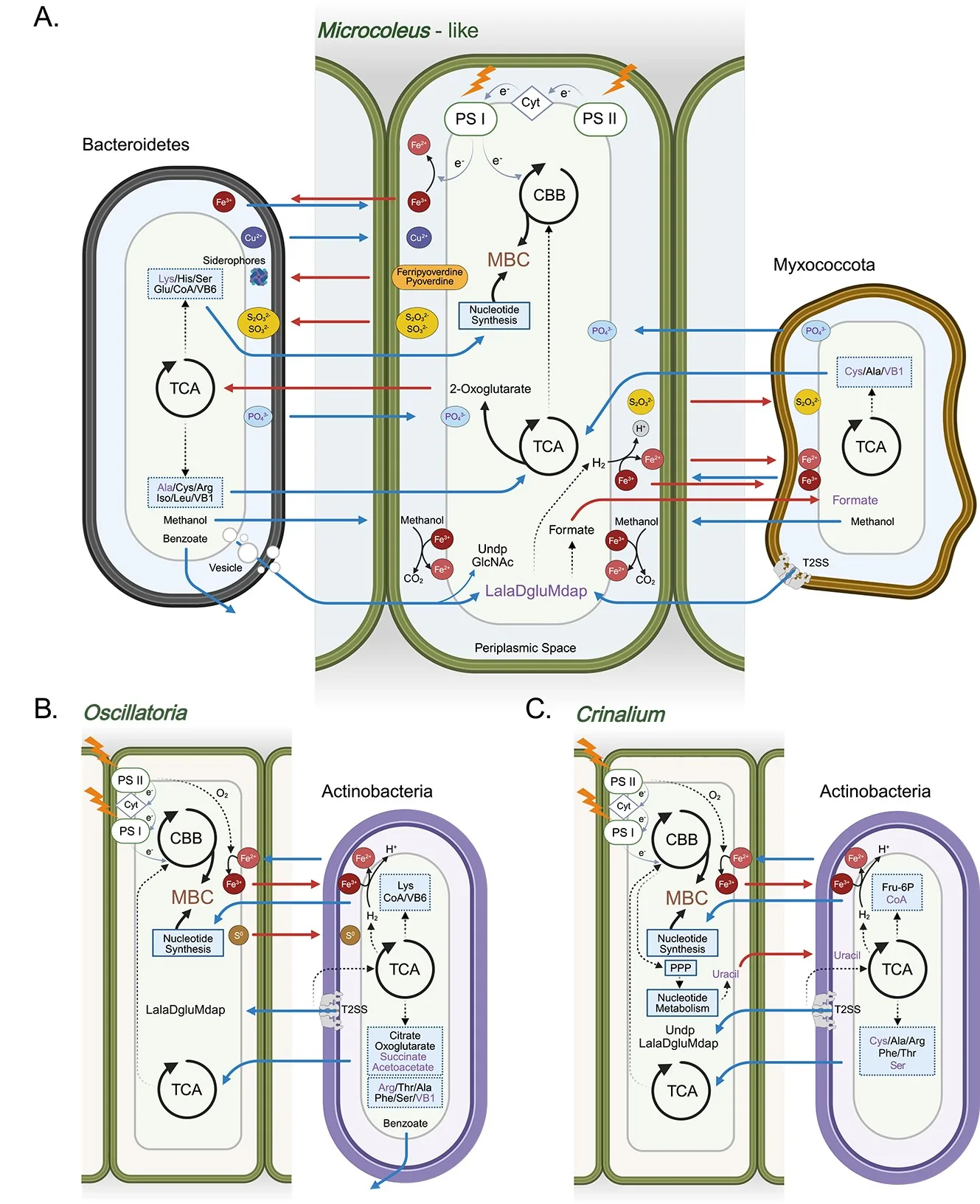
Metabolic syntrophy inferred from genome-scale modeling. Directed metabolite exchange maps predicted by *SMETANA* for dominant cyanobacteria-heterotrophic partnerships. Blue arrows indicate metabolites acquired by the focal cyanobacteria, and red arrows indicate metabolites supplied by cyanobacteria. (A) Predicted exchanges between *Microcoleus*-like populations and Bacteroidetes (development phase) or Myxococcota (seasonal phase). (B) Predicted exchange between *Oscillatoria* and Actinobacteria during the seasonal phase. (C) Metabolic exchange between *Crinalium* and Actinobacteria during the development phase. GlcNAc, N-acetyl-D-glucosamine-1-phosphate; LalaDgluMadap, L-alanine-D-glutamate-meso-2,6-diaminopimelate; Undp, undecaprenyl phosphate; VB_1_, thiamine; CBB, Calvin-Benson-Bassham cycle; TCA, tricarboxylic acid cycle.

Metabolite exchange prediction revealed that cyanobacteria acted primarily as net importers, receiving a broader range of compounds than they supplied. The inferred exchanges spanned cofactors, metal ions, inorganic nutrients, cell-wall precursors, amino acids, and organic carbon intermediates (Fig. S10). During the formation stages, the *Microcoleus*-Bacteroidetes pairing supported rapid proliferation through enhanced exchange of nucleic acid precursors (Fig. 6A). In the harsh seasonal phase, *Microcoleus*-like populations were instead linked with Myxococcota, with the predicted exchange of B vitamins and amino acids potentially contributing to the maintenance of core metabolism under stressful conditions, while formate and iron–sulfur transport was inferred to underpin redox balance. For the other dominant cyanobacteria, the development-phase association between *Crinalium* and Actinobacteria was characterized by reciprocal cycling of ribose-pentose intermediates alongside cyanobacterial acquisition of organic matter and amino acids (Fig. 6B). During the seasonal phase, the *Oscillatoria*-Actinobacteria interactome was highly asymmetric. *Oscillatoria* supply iron–sulfur clusters while receiving a wide array of metabolites, including essential amino acids, vitamins, tricarboxylic acid cycle intermediates, and ribose precursors (Fig. 6C). All cyanobacterial partners required exogenous iron and vitamins, and *Microcoleus*-like populations consistently showed dependence on heterotrophic phosphate provisioning. These modeling outputs demonstrate that syntrophic metabolite exchanges are stage-specific and may be fundamental to sustaining the metabolic activity and biomass of dominant cyanobacteria under the nutrient-limited, fluctuating conditions of dryland ecosystems.

### Mechanistic drivers of MBC accumulation

To explore the genomic basis of the observed successional patterns, we compared the functional gene content of the dominant cyanobacterial MAGs (Fig. S11). *Oscillatoria* MAGs exhibited a higher enrichment of genes associated with the S-strategy, whereas *Microcoleus*-like MAGs were significantly enriched in A-strategy genes, particularly those encoding specialized ABC transporters. With respect to phototrophic specialization (Table S1), *Crinalium* harbored high copy numbers of phycobilisome linker proteins (CpcG) and chemosensory proteins, while *Oscillatoria* was characterized by elevated copies of the D1 protein (PsbA), suggesting enhanced PSII repair capacity. Transport-system annotations (see Table S2) further revealed that both *Oscillatoria* and *Crinalium* possess diverse repertoires of transporters for central carbon metabolites. These genomic contrasts indicate niche partitioning among the dominant cyanobacterial lineages. Specifically, *Microcoleus*-like populations are optimized for rapid resource acquisition and growth during early colonization of oligotrophic substrates, whereas *Oscillatoria* and *Crinalium* are genomically equipped for more mature, nutrient-rich microenvironments, with *Oscillatoria* showing greater investment in stress resilience and *Crinalium* allocating more to light harvesting and chemotactic regulation.

**Figure 7:**
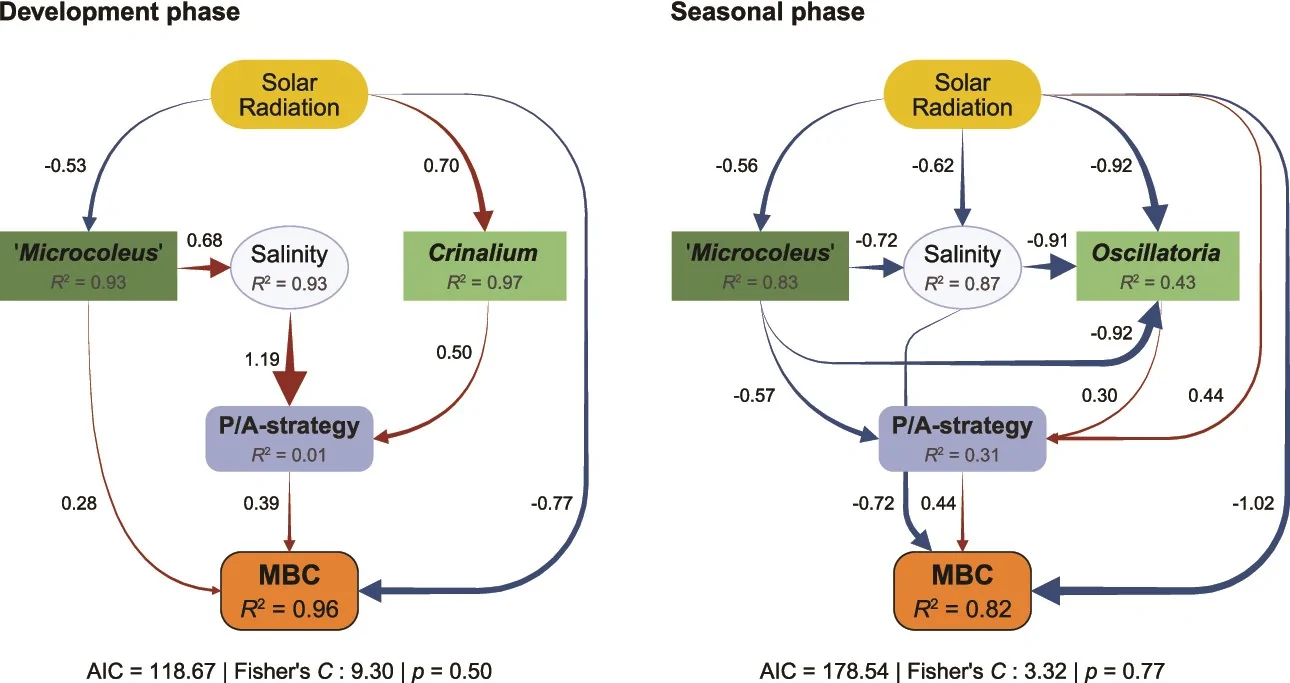
Environmental and biological linkages with microbial biomass carbon (MBC) accumulation. Piecewise structural equation models (pSEM) illustrate the causal cascades during the development phase (left) and seasonal phase (right). Red and blue arrows represent significant positive and negative standardized path coefficients (*P* < .05), respectively. Arrow thickness is proportional to the strength of the association. Solar radiation and salinity were associated with MBC indirectly through their effects on active cyanobacterial abundances and the community-level P/A-strategy ratio. *R*^2^ values denote the proportion of variance explained for each endogenous variable and MBC. Model fit was assessed using Fisher’s *C* test.

Random-forest modeling identified solar radiation and salinity as the most influential environmental predictors of MBC, alongside the relative active abundances of the three dominant cyanobacterial genera and the community-level P/A-strategy ratio (Fig. S12). pSEM further elucidated the cascades linking these variables (Fig. S13 and Fig. 7). We found that solar radiation exerted a direct negative effect on MBC, but its indirect effects were stage-specific. During the development phase, solar radiation was positively related to *Crinalium* abundance and an elevated P/A-strategy ratio. In contrast, it was negatively associated with *Microcoleus*-like transcriptional abundance, which was positively linked to salinity and subsequent A-strategy pathways. During the seasonal phase, solar radiation directly increased the P/A-strategy ratio, thereby facilitating MBC accumulation. *Oscillatoria* contributed positively to MBC via a positive link with the P/A-strategy ratio, whereas *Microcoleus*-like populations exerted a net negative effect, suppressing both *Oscillatoria* and the P/A-strategy ratio directly and indirectly through salinity-mediated effects. Collectively, these multi-omic and modeling results show that light intensity and soil salinity shape distributions of cyanobacterial composition and life-history strategies, which, in turn, affect variation in MBC accumulation. The transition from *Microcoleus*-dominated development to *Oscillatoria*-dominated seasonal stability reflects a functional handoff and shift from growth-centric resource acquisition toward stress-resilient production.

## Discussion

### Functional handoffs in biocrust succession

Cyanobacterial inoculation is increasingly used to accelerate biocrust formation and restore dryland soil functions [8, 9]. The success of such interventions is often inferred from whether the inoculated cyanobacteria persist and remain dominant after field introduction [57]. Our results indicate that inoculant persistence is pivotal but insufficient to explain how induced biocrusts accumulate MBC. The inoculated strain (*Konicacronema* sp. APE-1) remained among the most abundant primary producers throughout the circannual succession, yet multi-omics evidence reveals that taxonomic persistence and functional dominance were largely decoupled. *Microcoleus*-like populations exhibited higher activity during early biocrust development, whereas *Oscillatoria* became functionally prominent during the later seasonal phase.

This functional handoff coherently explains the observed carbon dynamics. During early development, increases in *F*v/*F*m, Chl *a*, and carbon-associated metabolic pathways marked the initial establishment of a phototrophic pioneer consortium. *Microcoleus*-like cyanobacteria were well-suited to this stage, stabilizing exposed sand surfaces, secreting EPS, and creating microhabitats for subsequent microbial colonization [10–12]. At this point, carbon accumulation is largely tied to surface engineering and rapid resource capture. During the seasonal phase, by contrast, *F*v/*F*m and Chl *a* declined while TOC and MBC remained significantly elevated. It suggests that carbon accumulation in biocrusts does not merely track stronger photosynthetic capacity. Rather, it reflects the holistic resilience of the photoautotrophic subcommunity in sustaining carbon fixation under seasonal environmental fluctuations, paralleling the seasonal phytoplanktonic assemblages observed in aquatic ecosystems [58, 59].

The seasonal phase was characterized by light-harvesting, cyclic electron transport, non-enzymatic antioxidant protection, and stress tolerance. These traits are ecologically meaningful in dryland biocrusts, where hydration-desiccation cycles, intense radiation, and temperature fluctuation strongly constrain cyanobacterial activity [60–62]. In this context, the increasing prominence of *Oscillatoria* populations is better understood not as a simple replacement of *Microcoleus*-like pioneers, but as a temporal division of functions among photoautotrophs. *Microcoleus*-like taxa drive early establishment and substrate capture, whereas *Oscillatoria*-like taxa help maintain functions as seasonal stress intensifies. This interpretation shifts the emphasis from single-strain dominance toward functional complementarity among cyanobacterial guilds, providing a community-level basis for the sustained carbon accumulation observed even as pioneer activity declines.

This division of functioning also clarifies how the taxonomic scope relates to its ecological conclusions. The *Microcoleus* complex has undergone substantial taxonomic revision [15, 16], and the inoculated strain is now assigned to *Konicacronema* sp. (Gomontiellales) [29, 30]. At the achievable taxonomic resolution, the omics data annotated as “*Microcoleus*” are therefore better interpreted as a *Microcoleus*-like functional signal rather than as exclusive evidence of activity from the inoculated strain, because indigenous close relatives may contribute to the same annotation. This boundary does not weaken our ecological conclusion. It even reinforces the reality of interpreting succession at the level of functional guilds rather than at the level of single taxa.

### Heterotrophic subsidies and resource acquisition

Coupled with activity handoffs within cyanobacteria across succession, metabolic interdependencies further help explain how that activity is sustained. We found that the dominant cyanobacteria are not merely primary producers but rather the metabolic core of a mutually supporting cyanosphere [63]. Genome-scale metabolic modeling predicted that cyanobacteria could draw amino acids, vitamins, cell-wall precursors, organic carbon, iron-bearing metabolites, and phosphate from their heterotrophic associates. Such inferred interdependency aligns with the recent recognition that metabolic exchange pervades natural microbial communities [17] and with prior demonstrations that the cyanosphere of *Microcoleus* spatially organizes nutrient trade around its filamentous bundle [14, 26]. These predicted subsidies provide a plausible mechanism for carbon pools to continue accumulating even as bulk photosynthetic indicators declined. More importantly, the predicted partnerships were not static but tracked biocrust development. During early establishment, *Microcoleus*-like populations were strongly associated with Bacteroidetes and were involved in mobilizing organic carbon, phosphorus, and sulfur [64]. As the seasonal phase began, dominant associations shifted toward *Oscillatoria*-Actinobacteria and *Crinalium*-Actinobacteria pairs, indicating a transition from substrate mobilization to maintaining activity through cofactor provision and nutrient recycling under stressful conditions. This reconfiguration matches the broader functional turnover of the cyanobacterial community.

These interaction dynamics are paralleled at the adaptation-strategy level. Modules linked to MBC were consistently enriched in acquisition-oriented pathways, especially ABC transporter systems, and the community-level P/A-strategy ratio aligned with MBC. These patterns highlight resource acquisition as a principal axis of carbon retention. In dryland biocrusts, microbial persistence demands more than carbon fixation. It also involves securing scarce nutrients, repairing stress-induced damage, and capitalizing on brief windows of favorable hydration [65, 66]. In this view, enriching acquisition traits is not an incidental metabolic feature but a coherent strategy for retaining biomass carbon when photosynthetic performance is significantly constrained [67]. It is consonant with microbial life-history theory, which treats resource acquisition, growth potential, and stress tolerance as context-dependent and pair-traded strategies [21, 23, 68], and with evidence that greater investment in acquisition can reshape growth-yield relationships under resource limitation [69].

Nonetheless, these inferences need to be validated and interpreted appropriately. For instance, co-occurrence networks of microbial taxa can arise from either shared environmental preferences or direct interactions [70], and *SMETANA*-based predictions remain contingent on MAG completeness, the fidelity of metabolic reconstruction, and the assumptions embedded in the underlying models [33, 56]. The exchanges reported here should accordingly be described as hypotheses for cyanospheric syntrophy, not as direct measurements of metabolite flux. However, these findings provide concrete and testable targets for future isotope-tracing, exometabolomic, and nanoscale secondary-ion mass spectrometry (NanoSIMS) analyses [71].

### Nutrient constraints on carbon accumulation

Although this study focuses on carbon accumulation, nitrogen and phosphorus trajectories are inseparable from biocrust maturation [72]. The results showed a steady increase in TN through the primary succession, and early functional profiles were enriched in assimilatory nitrogen pathways. Because *Konicacronema* sp., like most bundle-forming pioneers, is not a typical diazotroph, nitrogen accumulation largely reflects the recruitment and retention of nitrogen-fixing and nitrogen-cycling partners, a dependency well documented for the *Microcoleus*-associated cyanosphere [19, 26]. Although nitrogen fixation rates and *nifH* expression were not quantified in this study, the mechanism we consider most feasible is the construction of cyanobacterial niches. By stabilizing the surface, secreting EPS, retaining moisture, and exuding labile organic carbon, early pioneers establish localized, carbon-replete, microaerophilic microhabitats that favor diazotroph colonization [72, 73]. The establishment of nitrogen-cycling guilds by bundle-forming cyanobacteria lays the foundation for nitrogen retention and carbon-nitrogen exchange in biocrusts.

The results showed that phosphorus followed a contrasting trajectory. Soluble phosphate became depleted in the later phase, even as TOC and MBC continued to increase, suggesting that phosphorus availability was more limited as the biocrust matured. Several biochemical processes could underlie this decline, such as sequestration of phosphate into microbial biomass [74], immobilization within EPS-rich matrices [75], organomineral interactions [76], and the rising biological demand that accompanies the expansion of carbon and nitrogen pools [77]. These dynamics are consistent with both broader frameworks of terrestrial phosphorus limitation [20] and evidence that extracellular polymeric or alginate-like matrices can modulate phosphorus retention and bioavailability [78]. Phosphate depletion is therefore unlikely to be a mere by-product of edaphic physicochemical processes. It may signal a shift in the limiting resource, from the carbon- and colonization-limited conditions of early development toward a nutrient-balance-constrained regime in the later phase.

The nitrogen and phosphorus trends indicate that carbon accumulation in biocrusts cannot be understood in isolation from the wider nutrient stoichiometry. Carbon gain, nitrogen retention, and intensifying phosphorus demand are ultimately coupled, and the dominant constraint appears to migrate as the biocrust develops. Regardless of this phase transition, solely tracking soil organic carbon or chlorophyll content may fail to uncover the underlying constraints of long-term biocrust restoration.

### Implications for resilient biocrust restoration

Taken together, these findings suggest shifting biocrust restoration from maximizing the abundance of a single inoculant toward temporally complementary microbial consortia. Although a strong pioneer strain remains indispensable [79], particularly for the early stabilization of the mobile dune surface and EPS excretion, sustained carbon accumulation may require a broader functional toolkit than any one strain can provide. For instance, the *Microcoleus*-like bundle-forming clade initiates biocrust formation, while *Oscillatoria* or *Crinalium* taxa continue to maintain activity under seasonal stress, and heterotrophic partners undertake nutrient recycling and biosynthesis. This consortium-based strategy aligns with the recognized potential of cyanobacteria-heterotroph synthetic assemblages in restoration and biotechnology [80], as well as with previous findings that desert cyanobacteria and Actinobacteria orchestrate complementary stress-tolerance and nutrient-acquisition traits [28, 81].

Accordingly, the long-term success of an inoculation should be judged across several interdependent layers rather than by inoculant persistence, including survival of the introduced strain, activity of successive cyanobacterial guilds, recruitment of heterotrophic partners, dynamics of nutrient pools, and resulting accumulation of MBC. Our study offers an integrative framework for linking these layers in field-induced biocrusts, though its boundaries should also be considered. To be specific, the taxonomic resolution achievable here constrains strain-level inference of *Microcoleus*-like taxa turnover; co-occurrence networks and pSEM delineate plausible linkages without further direct examination; and genome-scale metabolic modeling forecasts potential exchanges rather than measured fluxes. Nevertheless, within these limits, a coherent picture emerges that the accumulation of biomass carbon in induced biocrusts is driven not by the dominance of a single pioneer, but by functional handoffs between cyanobacterial guilds and the heterotrophic subsidies that sustain them across shifting seasonal and nutrient constraints. It depicts a more robust route to enhance carbon storage and, ultimately, to resiliently stabilize degraded drylands through biocrust restoration.

## Supplementary Material

Supplementary_material_ycag254

## Data Availability

The 16S rRNA gene sequences, metagenome, and metatranscriptome datasets of induced biocrusts in this study are available in the NCBI database under project IDs PRJNA1434144, PRJNA1441662, and PRJNA1441815. The mass spectrometry proteomics data are deposited in iProX under dataset ID IPX0016056000.
