# Supplementary material for: Stage-specific cyanobacterial handoffs and heterotrophic subsidies underpin biocrust carbon accumulation
Source: ISME Commun. 2026 Sep 4;6(1):ycag254. doi: 10.1093/ismeco/ycag254 (PMC13602264; doi:10.1093/ismeco/ycag254)
Supplement: Supplementary file 1 [file supplementary_material_ycag254.zip › Supplementary_R1_ycag254.docx]

**Supplementary Information**

**Figure S1.** Phylogenetic analysis of high-abundance cyanobacterial ASVs was performed using *Gloeobacter* as the outgroup. The adjacent heatmap illustrates temporal dynamics in relative abundance across development and seasonal periods, where blue shading denotes low relative abundance and red indicates high relative abundance. Numeric values superimposed on heatmap cells represent exact quantitative measurements. **E** represents the early formation stage, **M** represents the middle formation stage, **L** represents the late formation stage, **Su** represents summer, **Au** represents autumn, **Wi** represents winter, and **Sp** represents spring.

**Figure S2.** Community structural characteristics at the genus level based on metaproteomics. Different colors represent different taxonomic groups. **E** represents the early formation stage, **M** represents the middle formation stage, **L** represents the late formation stage, **Su** represents summer, **Au** represents autumn, **Wi** represents winter, and **Sp** represents spring.

**Figure S3.** The community structural characteristics at the genus level based on metaproteomics. Different colors represent different taxonomic groups. **E** represents the early formation stage, **M** represents the middle formation stage, **L** represents the late formation stage, **Su** represents summer, **Au** represents autumn, **Wi** represents winter, and **Sp** represents spring.

**Figure S4.** Proportion of predominant life-history strategies among high-abundance species within the three co-occurrence network clusters during (**A**) development phase and (**B**) seasonal phase at the ***metatranscriptomic*** level, summarized based on metabolic pathways from the KEGG database. Strategies include: A-strategy (resource acquisition), P-strategy (growth potential), and S-strategy (stress tolerance).

**Figure S5.** Proportion of predominant life-history strategies among high-abundance species within the three co-occurrence network clusters during (**A**) development phase and (**B**) seasonal phase at the ***metaproteomic*** level, summarized based on metabolic pathways from the KEGG database. Strategies include: A-strategy (resource acquisition), P-strategy (growth potential), and S-strategy (stress tolerance).

**Figure S6.** Transported substances with up-regulated expression at the metaproteomic level during the development and seasonal periods. **E** represents the early formation stage, **M** represents the middle formation stage, **L** represents the late formation stage, **Su** represents summer, **Au** represents autumn, **Wi** represents winter, and **Sp** represents spring.

**Figure S7**. Regression analysis results of the changes in microbial biomass carbon (MBC) and life-history strategies during the development and seasonal periods.

**Figure S8**. Phylogenetic tree constructed jointly from the target MAG (*s_metawrap_bin.8*) and genomes of sheath-forming *Microcoleus*. The target MAG is indicated in black, while the sheath-forming *Microcoleus* genomes are shown in yellow.

**Figure S9**. Correlation between the *PhyloMint* complementarity index (MI*_complementarity_*) and phylogenetic distance (PD); the fitted curve was constructed using a generalized linear model (GLM), with the shaded area representing the 95% confidence interval. The correlation was statistically tested using Kendall’s rank correlation test (two-sided). The density distribution plots describe the statistical characteristics of the interactions, with the density curves above and to the right displaying the distribution of interactions along the PD axis and the *PhyloMint* complementarity index, respectively.

**Figure S10**. Composition of dominant Actinobacteria species during the development and seasonal stages (**A**), and exchanged metabolites between metabolically complementary species (**B** and **C**). In panel A, different colors represent different families of Actinobacteria dominant in the development and seasonal stages. The bar charts on the left side of panels B and C use different colors to indicate distinct categories of metabolites; the labels a, b, c, and d below denote different interacting species pairs. The red heatmap represents the predicted index of metabolite exchange.

**Figure S11**. Comparison of copy numbers for *Microcoleus* and *Oscillatoria* across the A-, P-, and S-strategy pathways. Here, M represents *Microcoleus*, and O represents *Oscillatoria*. The black text at the top indicates the P-strategy, representing growth potential; the yellow text below indicates the S-strategy, representing stress tolerance; and the blue text on the right indicates the A-strategy, representing resource acquisition.

**Figure S12.** Major predictors of microbial biomass carbon growth during the crust development and seasonal stages. Percent increase in mean squared error (%IncMSE) based on random forest analysis. Different colors on the left represent different categories: red indicates species (genus level), yellow represents climatic parameters, blue denotes crust physicochemical properties, and black corresponds to life history strategies.

**Figure S13.** Hypothesized a priori SEM model of the effects in the direct and indirect drivers of microbial biomass carbon (MBC) dynamics. The predictors include solar radiation, edaphic properties (EC), life-history strategies (P/A-strategy ratio), and important dominated cyanobacteria. The serial numbers in the figure correspond to detailed references that have previously proven the paths (see below).

**Path: ①**

Ohad I, et al. 2010. Light-induced changes within Photosystem II protects *Microcoleus* sp. in biological desert sand crusts against excess light. ***PLoS ONE***, 5:e11000. https://doi.org/10.1371/journal.pone.0011000

**Path: ②**

Zeng ZJ, et al. 2024. An improved cracked soil evaporation model accounting for solar radiation and wind effect. ***Computers and Geotechnics***, 174:106598. https://doi.org/10.1016/j.compgeo.2024.106598

Shokri N and Or D. 2011. Quantitative analysis of evaporation dynamics from porous media. ***Water Resources Research***, 47:W09513. https://doi.org/10.1029/2010WR009879

**Path: ③⑧**

Anton FP, et al. 1985. Regulation of growth and photosynthesis by *Oscillatoria agardhii* grown with a light/dark cycle, ***FEMS Microbiology Ecology***, 1(2):97-102. https://doi.org/10.1111/j.1574-6968.1985.tb01136.x

Sivonen K. 1990. Effects of light, temperature, nitrate, orthophosphate, and bacteria on growth of and hepatotoxin production by *Oscillatoria agardhii* strains. ***Appl Environ Microbiol***, 56(9):2658-66. https://doi.org/10.1128/aem.56.9.2658-2666.1990

**Path: ④**

Jan Z, et al. 2023. Cyanobacterial strains reclaimed induced-salinity stress attributes and improved the physicochemical properties of induced-saline soil. ***Acta Ecologica Sinica***, 43:1074-1079. https://doi.org/10.1016/j.chnaes.2023.03.003

**Path: ⑤**

Wangwibulkit S, et al. 2017. Effects of salinity and pH on the growth of blue-green algae, *Oscillatoria* sp. and *Microcystis* sp., isolated from Pacific white shrimp (*Litopenaeus vannamei*) ponds. ***Journal of Fisheries and Environment***, 32(1):1-9. https://li01.tci-thaijo.org/index.php/JFE/article/view/81394

**Path: ⑥**

Nelson C, et al. 2022. Spatial self-segregation of pioneer cyanobacterial species drives microbiome organization in biocrusts. ***ISME Communications***, 2:114. https://doi.org/10.1038/s43705-022-00173-3

**Path: ⑦**

Nelson C, et al. 2021. A symbiotic nutrient exchange within the cyanosphere microbiome of the biocrust cyanobacterium, Microcoleus vaginatus. ***ISME***, 15:282-292. https://doi.org/10.1038/s41396-020-00761-3

Lan S, et al. 2022. Cyanobacterial persistence and influence on microbial community dynamics over 15 years in induced biocrusts. ***Environmental Microbiology***, 24(1):66-81. https://doi.org/10.1111/1462-2920.15853

**Path: ⑨**

Jahn M, et al. 2018. Growth of cyanobacteria is constrained by the abundance of light and carbon assimilation proteins. ***Cell Reports*** 25:478-486.e8. https://doi.org/10.1016/j.celrep.2018.09.062

**Path: ⑩**

Mavi MS and Marschner P. 2013. Salinity affects the response of soil microbial activity and biomass to addition of carbon and nitrogen. ***Soil Research***, 51:68-75. https://doi.org/10.1071/SR12191

Jia B, et al. 2024. Salinity decreases the contribution of microbial necromass to soil organic carbon pool in arid regions. ***Science of the Total Environment***, 930:172786. https://doi.org/10.1016/j.scitotenv.2024.172786

**Path: ⑪**

Malik AA, et al. 2019. Soil microbial communities with greater investment in resource acquisition have lower growth yield. ***Soil Biology and Biochemistry***, 132:36-39. https://doi.org/10.1016/j.soilbio.2019.01.025

**Path: ⑫**

Johnson D, et al. 2003. UV-B radiation and soil microbial communities. ***Nature***, 423:137-138. https://doi.org/10.1038/423138a

**Supplemental Tables**

**Table S1.** Copy numbers of key functional genes across different cyanobacterial genera.

**Table S2.** Variations in transporter copy numbers across different cyanobacterial genomes based on substrate specificity.
